# Uhlmann number in translational invariant systems

**DOI:** 10.1038/s41598-019-45546-9

**Published:** 2019-06-24

**Authors:** Luca Leonforte, Davide Valenti, Bernardo Spagnolo, Angelo Carollo

**Affiliations:** 1 Department of Physics and Chemistry - Emilio Segrè, Group of Interdisciplinary Theoretical Physics, University of Palermo, Viale delle Scienze, Ed. 18, I-90128 Palermo, Italy; 2 Istituto di Biomedicina ed Immunologia Molecolare (IBIM) “Alberto Monroy”, CNR, Via Ugo La Malfa 153, I-90146 Palermo, Italy; 3 Radiophysics Department, Lobachevsky State University of Nizhni Novgorod, 23 Gagarin Avenue, Nizhni Novgorod, 603950 Russia; 4 Istituto Nazionale di Fisica Nucleare, Sezione di Catania, Via S. Sofia 64, I-95123 Catania, Italy

**Keywords:** Topological insulators, Theoretical physics

## Abstract

We define the *Uhlmann number* as an extension of the Chern number, and we use this quantity to describe the topology of 2D translational invariant Fermionic systems at finite temperature. We consider two paradigmatic systems and we study the changes in their topology through the *Uhlmann number*. Through the linear response theory we link two geometrical quantities of the system, the *mean Uhlmann curvature* and the *Uhlmann number*, to directly measurable physical quantities, i.e. the dynamical susceptibility and the dynamical conductivity, respectively. In particular, we derive a non-zero temperature generalisation of the Thouless-Kohmoto-Nightingale-den Nijs formula.

## Introduction

The discovery of topological ordered phases (TOP) has attracted an ever growing interest from the very outset^1^, partly due to the number of fascinating phenomena connected to it, such as topologically protected edge excitations^2^, quantised current in insulating systems^3–8^, bulk excitations with exotic statistics^9–11^. A relevant subclass of TOP are the so called symmetry-protected TOP, which have been extensively studied and classified thoroughly, according to a set of topological invariants^12–15^. The above classification relies on the assumption that the relevant features of a topological quantum system are fundamentally captured by the system zero-temperature limit, i.e. by the properties of its pure ground state. However, the fate of these topological ordered phases remains unclear, when a mixed state is the faithful description of the quantum system, either because of thermal equilibrium, or due to out-of-equilibrium conditions^16–23^. Over the last few years, different attempts have been done to reconcile the above topological criteria with a mixed state configuration^24–34^. The recent success of the Uhlmann approach^35^ in describing the topology of 1D Fermionic systems^26,27^, remains in higher dimensions^28^ not as straightforward^29^. Moreover, the importance of this approach and its relevance to directly observable physical quantities still remains an interesting open question.

In this work, we propose to study 2D topological Fermionic systems, at finite temperature, by means of a new set of geometrical tools derived from the Uhlmann approach^35^, and more specifically from the *mean Uhlmann curvature* (MUC)^36,37^. We study 2D-topological insulators (TIs), whose topological features are captured by the Chern number^15^. As with many other topological materials, these systems may host gapless edge excitations, whose presence characterises the onset of a non-trivial topological phase^38^.

For translational invariant models, one can define the Chern number as ${\rm{Ch}}=\frac{1}{2\pi }{\iint }_{BZ}{F}_{xy}^{B}{d}^{2}{\bf{k}}$, i.e. the integral over the Brillouin zone (BZ) of the Berry curvature ${F}_{xy}^{B}$. The Ch is always an integer and it is the topological invariant that characterises the zero-temperature phase of the system we are interested in. In order to study these models at finite temperature one should find a way to generalise the Chern number to a mixed state scenario. However, a direct generalisation of the Chern number via the Uhlmann approach leads to a trivial topological invariant. In this work, we construct a quantity, *the Uhlmann number n*_*U*_, through the MUC. Stricktly speacking, this quantity is not a topological invariant, but it provides a faithful description of topological and geometrical properties of the systems with respect to temperature changes. We apply these concepts to two paradigmatic models of TI, the QWZ model^39^ and a TI with high Chern number^40,41^, and explicitly derive the dependence of the Uhlmann number on temperature. Beyond their mathematical and conceptual appeal, we show that the MUC and Uhlmann number are related to quantities directly accessible to experiments, namely, the susceptibility to external perturbations and the transverse conductivity.

## Results

### Susceptibility and mean Uhlmann curvature

The Uhlmann approach to geometric phase of mixed states allows to define a mixed state generalization of the Berry curvature, the *mean Uhlmann curvature* (MUC). The MUC can be defined as the Uhlmann geometrical phase over an infinitesimal loop (see section Methods)

1 $${{\mathscr{U}}}_{\mu \nu }\,:\,=\mathop{\mathrm{lim}}\limits_{{\delta }_{\mu }{\delta }_{\nu }\to 0}\frac{{\phi }^{U}[\gamma ]}{{\delta }_{\mu }{\delta }_{\nu }}\mathrm{.}$$

The MUC is a geometrical quantity, whose definition relies on a rather formal definition of holonomies of density matrices. In spite of its abstract formalism, the MUC has interesting connections to a physically relevant object which is directly observable in experiments, the susceptibility. By using the linear response theory, we can indeed relate the MUC to the dissipative part of the dynamical susceptibility. Indeed, one can consider the most general scenario of a system with a Hamiltonian ${ {\mathcal H} }_{0}$, perturbed as follows

2 $$ {\mathcal H} ={ {\mathcal H} }_{0}+\sum _{\mu }{\hat{O}}_{\mu }{\lambda }_{\mu },$$

where $\{{\hat{O}}_{\mu }\}$ is a set of observables of the system, and $\{{\lambda }_{\mu }\}$ is the corresponding set of perturbation parameters. Then, we show (see section Methods) that for a thermal state, the dissipative part of the dynamical susceptibility ${\chi }_{\mu \nu }^{^{\prime\prime} }(\omega ,\beta )$ is related to the MUC as follows

3 $${{\mathscr{U}}}_{\mu \nu }=\frac{i}{\pi \hslash }{\int }_{-\infty }^{+\infty }\frac{d\omega }{{\omega }^{2}}{\tanh }^{2}(\frac{\omega \beta }{2}){\chi }_{\mu \nu }^{^{\prime\prime} }(\omega ,\beta ),$$

where the set of perturbations $\{{\lambda }_{\mu }\}$ in (2) plays the role of the parameters in the derivation of ${{\mathscr{U}}}_{\mu \nu }$, and where *β* := 1/*k*_*B*_*T*, is the inverse of the temperature. Moreover, by means of the fluctuation-dissipation theorem^42^, one can also derive a further expression for Eq. (3) in terms of the dynamical structure factor, ${S}_{\mu \nu }(\omega ,\beta )={\int }_{-\infty }^{+\infty }dt{e}^{i\omega t}{S}_{\mu \nu }(t)$, (i.e. the Fourier transform of the correlation matrix ${S}_{\mu \nu }(t)=\langle {\hat{O}}_{\mu }(t){\hat{O}}_{\nu }\mathrm{(0)}\rangle$) namely

4 $${{\mathscr{U}}}_{\mu \nu }=\frac{i}{2\pi \hslash }{\int }_{-\infty }^{+\infty }\frac{d\omega }{{\omega }^{2}}{\tanh }^{2}(\frac{\omega \beta }{2})({S}_{\mu \nu }(\omega ,\beta )-{S}_{\nu \mu }(\,-\,\omega ))\mathrm{.}$$

Equations (3) and (4) provide a means to explore experimentally the geometrical properties of physical systems via the dissipative part of the dynamical susceptibility, and the imaginary part of the (off-diagonal)-dynamical structure factor.

Beyond its geometrical meaning, one can also show that the MUC has profound interpretation in terms of quantum multi-parameter estimation theory^36,37,43–45^. Indeed, the uncertainty in the estimation of a set of parameters $\{{\lambda }_{\mu }\}$ of a physical system is lower bounded by the Cramer-Rao (CR) bound^46–48^, i.e. ${\rm{Cov}}(\hat{\lambda })\ge {J}^{-1}$, where *J* is the quantum Fisher information matrix, whose elements are ${J}_{\mu \nu }=\frac{1}{2}{\rm{Tr}}[\rho \{{L}_{\mu },{L}_{\nu }\}]$, and ${\rm{Cov}}(\hat{\lambda })$ is the covariance matrix, which quantifies the uncertainty on $\{{\lambda }_{\mu }\}$. Both in a *classical multi-parameter* and in a *quantum single-parameter* estimation problem, the CR bound is always tight. However, in the *quantum multi-parameter* case, the CR bound may not be saturated, due to a manifestation of the uncertainty principle, known as *incompatibility condition*^43–45^. Such an incompatibility is quantified by the MUC^36,43^, which signals whether the estimation of a set of parameters is hindered by the inherent quantum nature of the underlying physical system.

Thanks to Eq. (3) we see that if the perturbations are longitudinal, so that they affect only the expectation value of the correspondent operator, then the MUC must be zero, and so the two parameters are *compatible*. On the converse, a transverse susceptibility signals the presence of an incompatibility which emerges from to the quantum nature of the physical system.

### Electrical conductivity and *n*_*U*_

The geometrical interpretations of the MUC as a generalisation of the Berry curvature and its connection to physically accessible quantities are quite desirable features. One may wonder whether these properties may be used to construct a physically appealing finite-temperature generalisation of a topological invariant, i.e. the Chern number.

The Chern number, ${\rm{C}}{\rm{h}}=\frac{1}{2\pi }{\int }_{BZ}{F}_{xy}^{B}d{k}_{x}d{k}_{y}$, is the invariant that characterises the topology of the bands in 2D translational invariant systems, where ${F}_{xy}^{B}$ is the Berry curvature. A natural finite temperature generalisation of the Ch can be constructed out of MUC, ${{\mathscr{U}}}_{\mu \nu }({\bf{k}})$ (see section Methods), as

5 $${n}_{U}=\frac{1}{2\pi }{\int }_{BZ}{{\mathscr{U}}}_{\mu \nu }d{k}_{\mu }d{k}_{\nu }\mathrm{.}$$

*n*_*U*_ is clearly a finite temperature generalisation of the Chern number, to which it converges in zero temperature limit. One should notice, however, that *n*_*U*_ is not itself a topological invariant, as it is not always an integer. Nevertheless, it provides a measure of the geometrical properties of the system and, above all, *n*_*U*_ posses quite remarkable connections to quantities which are readily accessible in experiments.

Indeed, consider a translational invariant 2D Fermionic system. In the quasi-momentum representation, the Hamiltonian reads ${ {\mathcal H} }_{0}={\sum }_{{\bf{k}}\in BZ} {\mathcal H} ({\bf{k}})$. When the system is perturbed by a time-dependent homogeneous electric field, one can show that the dissipative part of the dynamical transversal conductivity is directly linked to the Uhlmann number (Eq. (5)) via the following expression (see section Methods)

6 $$\frac{1}{\pi }{\int }_{-\infty }^{+\infty }\frac{d\omega }{\omega }{\tanh }^{2}(\frac{\hslash \omega \beta }{2}){\sigma }_{xy}^{^{\prime\prime} }(\omega ,\beta )=-\,\frac{{e}^{2}}{2\pi \hslash }{n}_{U}\mathrm{.}$$

From the definition and the properties of ${\sigma }_{\mu \nu }^{^{\prime\prime} }(\omega ,\beta )$ (see section Methods), Eq. (6) can be rewritten as

7 $${n}_{U}\frac{{e}^{2}}{2\pi \hslash }=-{\int }_{-\infty }^{+\infty }d\omega {\tilde{\sigma }}_{xy}(\omega ,\beta ){K}_{\beta }(\omega ),$$

where ${\tilde{\sigma }}_{xy}(\omega ,\beta )\,:\,={\rm{Re}}[{\sigma }_{xy}(\omega ,\beta )-{\sigma }_{yx}(\omega ,\beta )]\mathrm{/2}$ is the real, antisymmetric part of the transverse conductivity, and the kernel *K*_*β*_(*ω*) is a probability density function over the frequency domain $\omega \in {\mathbb{R}}$, that tends to the Dirac *δ*(*ω*) in the zero temperature limit. The expression in Eq. (7) is clearly a finite-temperature extension of the famous Thouless-Kohmoto-Nightingale-den Nijs (TKNN) formula^4^, i.e.

8 $${\sigma }_{xy}=-\,{\rm{Ch}}\frac{{e}^{2}}{h},$$

which connects the transversal conductivity of a topological insulator to the Chern number. In the same spirit, Eqs (6) and (7) provide a relation, valid at any temperatures, between the transversal conductivity and the geometrical properties of the band structure described by *n*_*U*_. A relevant difference between Eqs (7) and (8) is that the latter involves an average of the dynamical conductivities on a frequency band peaked around *ω* = 0, with a width Δ*ω* ∝ 1/*ħβ*. Nevertheless, Eqs (6) and (7) provide the operational means to probe experimentally the geometrical properties of the system at any finite temperature.

Moreover, combining Eqs (3) and (6) we get

9 $${{\mathscr{U}}}_{{E}_{x}{E}_{y}}=-\frac{{e}^{2}}{{\hslash }^{2}}2\pi {n}_{U},$$

where ${{\mathscr{U}}}_{{E}_{x}{E}_{y}}$ is the MUC, in which, two orthogonal components *E*_*x*_ and *E*_*y*_ of the electric field take the role of the parameters {*λ*_*μ*_} with respect to which the MUC is calculated. Hence, equation (9) links the topology of the system to the MUC (see section Methods), derived with respect to physically accessible external parameters, namely the electric fields. Interestigly, one can also show^36,37,43^ that the MUC has a very profound interpretation in terms of quantum estimation theory. Namely, ${{\mathscr{U}}}_{\mu \nu }$ marks the *incompatibility* of two parameters *λ*_*μ*_ and *λ*_*ν*_, in the sense specified in^36,37,43^, when these parameters needs to be evaluated simultaneously by any quantum multi-parameter estimation protocol. This incompatibility is a manifestation of the quantum uncertainty-principle, arising from the inherent quantum nature of the underlying physical system. When applied to Eq. (9), this argument links the presence of a non-trivial topology in the system to an *incompatibility* between the orthogonal components *E*_*x*_ and *E*_*y*_ of the electric field, in a quantum estimation protocol.

In the following two subsections we will apply some of the general considerations described so far to two archetypical models of 2D topological insulator.

### A two-dimensional topological insulator with high Chern number

A prototypical example of a 2D Chern insulator is a model that was first proposed by D. Sticlet *et al*.^40^. This is a topological insulator of Fermions lying on the vertices of a triangular lattice. Each Fermion carry a two-dimensional internal degree of freedom. By tweaking the interaction parameters, this model can be tuned to up to five different topological phases. Here, we consider Sticlet’s model with the following parametrisation

10 $$ {\mathcal H} =\sum _{ij}[{c}_{i+\mathrm{1,}j}^{\dagger }({t}_{1}{\sigma }_{1}+i{t}_{3}{\sigma }_{3}){c}_{i,j}+{c}_{i,j+1}^{\dagger }({t}_{1}{\sigma }_{2}+i{t}_{3}{\sigma }_{3}){c}_{i,j}+{c}_{i+\mathrm{1,}j+1}^{\dagger }{t}_{2}{\sigma }_{3}{c}_{i,j}+{\rm{H}}{\rm{.c}}{\rm{.}}]\mathrm{.}$$

The Pauli matrices describe the internal degree of freedom and *t*_*i*_ is a hopping amplitude coupling nearest neighbour Fermions with different orbitals. In the momentum representation the Hamiltonian reads

11 $$H({\bf{k}})=2\{\cos ({k}_{x}){\sigma }_{1}+\,\cos ({k}_{y}){\sigma }_{2}+[{t}_{2}\,\cos ({k}_{x}+{k}_{y})+\,\sin ({k}_{x})+\,\sin ({k}_{y})]{\sigma }_{3}\},$$

where we have set *t*_1_ = *t*_3_ = *t* = 1, and all the energies are scaled with respect to these parameters. The topological phases at zero temperature are characterised by the Chern number, whose value, as a function of *t*_2_, reads as

12 $${\rm{Ch}}=\{\begin{array}{ll}+2, & {\rm{if}}\,{t}_{2} < -\,2\\ +1, & {\rm{if}}-\,2 < {t}_{2} < 0\\ -1, & {\rm{if}}\,0 < {t}_{2} < 2\\ -2, & {\rm{if}}\,{t}_{2} > 2.\end{array}$$

**Figure 1:**
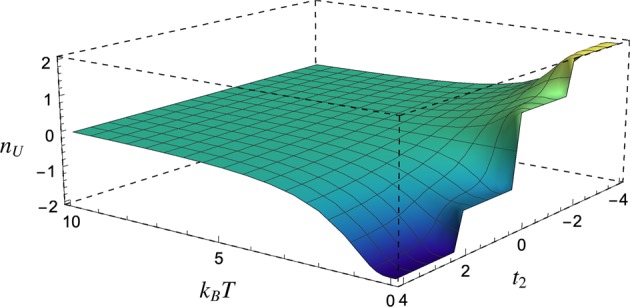
The graph shows how *n*_*U*_ changes for a topological insulator, with high Chern number, as a function of the temperature and the hopping term *t*_2_.

Notice that this model carries a non-trivial zero-temperature topological phase (i.e. Ch ≠ 0) for the whole parameter space. We consider the system in a thermal Gibbs state and we numerically calculate the Uhlmann number (see Eq. (19)), whose values are graphically represented as a function of *t*_2_ and temperature *T* in Fig. 1. As expected, the *n*_*U*_ correctly describes the topological phase transition at zero temperature. For high temperatures, the behaviour of *n*_*U*_ shows a typical cross-over transition, without any criticality between different regions^27,29,31^. One can observe a smooth monotonic vanishing of *n*_*U*_ as the temperature increases.

**Figure 2:**
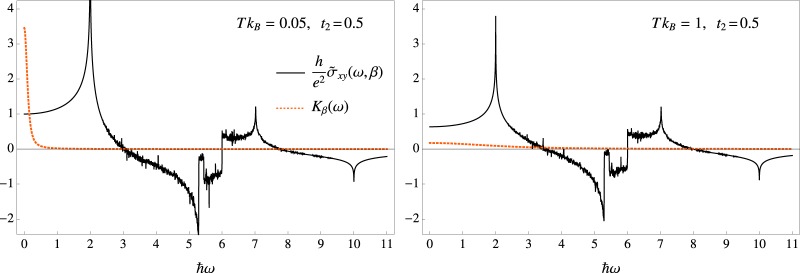
The graphs display the dependence of ${\tilde{\sigma }}_{xy}(\omega )$ (black, solid line) and the Kernel ${K}_{\beta }(\omega ,\beta )$ (orange, dashed line), on the frequency *ω* for $T{k}_{B}=0.05$ and $T{k}_{B}=1$ and parameter ${t}_{2}=0.5$. The transverse conductivity ${\tilde{\sigma }}_{xy}(\omega )$ displays van Hove singularities across the single particle spectrum of the model, which, for *t*_2_ = 0.5, ranges from *ω* = 2 to *ω* = 10. ${K}_{\beta }(\omega )$ is centered around *ω* = 0 and approximately non-vanishing only below the frequency bandwidth of ${\rm{\Delta }}\omega \simeq \frac{10}{\hslash \beta }$.

In order to grasp a better understanding of the relation, predicted by Eq. (7), between *n*_*U*_ and the real conductivity, we consider the behaviour of ${\tilde{\sigma }}_{xy}$ and *K*_*β*_ with respect to frequency and temperature. Figure (2) graphically shows ${\tilde{\sigma }}_{xy}$ and the probability density function *K*_*β*_ as a function of *ω* for two temperatures, $T{k}_{B}=0.1$ and $T{k}_{B}=2$, and for *t*_2_ = 0.5 (corresponding to a zero-temperature Ch = −1). As expected, for small temperatures the real transverse conductivity approaches the value ${\tilde{\sigma }}_{xy}\mathrm{(0)}\frac{\hslash }{{e}^{2}}\simeq -\,Ch=1$. The figure shows the distinctive dependence of the conductivity on the density of states (see Eq. (33)), featuring van Hove singularities across the single particle frequency band. The latter, for the chosen parameter *t*_2_ = 0.5, extends from *ω* = 2 to *ω* = 10. For the same values of the parameters, the shape of the probability density function *K*_*β*_ shows strong dependence on temperature. The distribution is sharply peaked around the static conductivity for small values of temperature, and broadens up for higher values of *T*. This explains, on the one hand, the strong dependence of *n*_*U*_ on temperature, and, on the other hand, the rather weak dependence of *n*_*U*_ on the dynamical conductivity even for relatively small values of the frequencies. As a consequence, the singular features of ${\tilde{\sigma }}_{xy}$ are not observable in *n*_*U*_, because they are either neglected by *K*_*β*_, for small values of *T*, or washed out in the averaging process, as *T* grows.

### QWZ model

In this section we consider the QWZ model, introduced by Qi, Wu and Zhang^39,49^ as an archetypical example of topological insulator. The QWZ Hamiltonian is constructed from the Rice-Mele model, where time is promoted to a spatial dimension. This system provides the simplest example of an anomalous quantum Hall system. The QWZ is a model of Fermions on a square lattice, with a two-dimensional orbital degrees of freedom per site, and its Hamiltonian is given by

13 $$ {\mathcal H} =J\sum _{ij}[{c}_{i+\mathrm{1,}j}^{\dagger }(\frac{{\sigma }_{z}+i{\sigma }_{x}}{2}){c}_{i,j}+{c}_{i,j+1}^{\dagger }(\frac{{\sigma }_{z}+i{\sigma }_{y}}{2}){c}_{i,j}+H\mathrm{.}c\mathrm{.}]+uJ\sum _{ij}{c}_{i,j}^{\dagger }{\sigma }_{z}{c}_{i,j}^{\dagger },$$

where *σ*_*i*_ are the Pauli matrix and *J* fixes the global energy scale, and for simplicity we set *J* = 1. The single-particle Hamiltonian in the quasi-momentum representation is

14 $$H({\bf{k}})=\{\sin \,{k}_{x}{\sigma }_{x}+\,\sin \,{k}_{y}{\sigma }_{y}+[u+\,\cos \,{k}_{x}+\,\cos \,{k}_{y}]{\sigma }_{z}\},$$

where the *σ*_*i*_ act on the orbital degrees of freedom. The topological phases of the model at *T* = 0 are characterised by the following Chern numbers as a function of *u*

15 $${\rm{Ch}}=\{\begin{array}{ll}0, & {\rm{if}}\,u < -\,2\\ 1, & {\rm{if}}-\,2 < u < 0\\ -1, & {\rm{if}}\,0 < u < 2\\ 0, & {\rm{if}}\,u > 2.\end{array}$$

**Figure 3:**
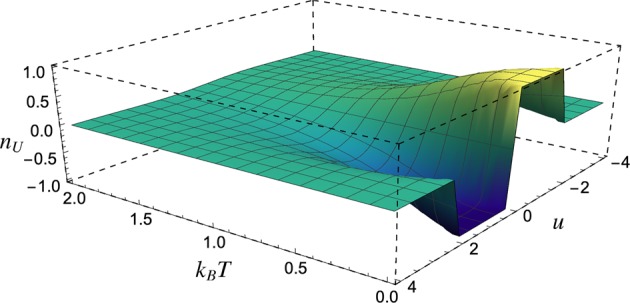
QWZ model: Uhlmann number behaviour as a function of temperature *T* and the parameter u.

For topological non-trivial regions, Ch = ±1, the system presents chiral edges states, as in the integral quantum Hall effect. We assume a thermal Gibbs state, and numerically calculate the Uhlmann number (see Eq. (19)), whose values are graphically represented in Fig. 3. As expected, the *n*_*U*_ correctly describes the topological phase transition at zero temperature. For high temperatures, the behaviour of *n*_*U*_ shows a typical cross-over transition, without any criticality between different regions. One can observe a smooth vanishing of *n*_*U*_ as the temperature increases.

**Figure 4:**
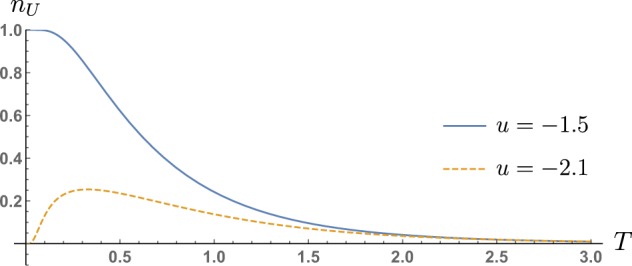
QWZ model: Uhlmann number behaviour as a function of temperature *T* for two different values of the parameter *u*, namely *u* = −1.5 and *u* = −2.1.

By fixing *u* in a specified phase, one can see two different dependencies of *n*_*U*_ as temperature increases. In a non-trivial topological phase, e.g. when Ch = ±1, we see that *n*_*U*_ vanishes monotonically (see the blue solid line in Fig. 4). On the other hand, one can see a peculiar non-monotonic behaviour of *n*_*U*_ in the trivial phase, for values of the parameter *u* in the close proximity of the critical point (see the dashed orange line in Fig. 4).

This can be interpreted as a thermal activation of the topological property of the system. Indeed, in a phase, which is trivial at zero temperature, there may be a range of temperatures for which the geometrical properties of the bands show non-trivial values. This can be explained by a thermal transfer of population from the valence to the conduction band, in the regions of the Brillouin zone in which the gap is smaller. These are the regions which contribute the most to the Uhlmann curvature, overall providing a non-trivial net value of the Uhlmann number. The closer the system is to a critical point, (for example for *u* → −2^−^ in the QWZ model), the more pronounced this effect is. This is due, on the one hand, by the narrowness of the gap which allows the valence band in this region of the BZ to be populated for relatively small values of *T*, and on the other hand, by the nearly divergent behaviour of the Berry curvature in the vicinity of the gap.

**Figure 5:**
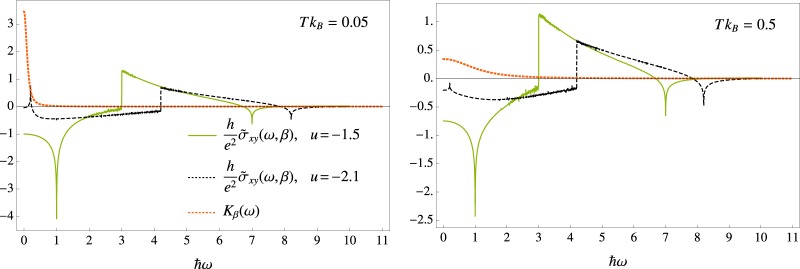
The graphs plots the dependence of real transverse conductivity ${\tilde{\sigma }}_{xy}(\omega ,\beta )$ (in units of ${e}^{2}/h$) and the Kernel ${K}_{\beta }(\omega )$, on the frequency *ω* for two temperatures, $T{k}_{B}=0.05$ and $T{k}_{B}=0.5$. One can appreciate in both plots the presence of van Hove singularities. In particular, one can observe the appearance of a singularity at *ω* = Δ, i.e. the band-gap of the model, which is $\hslash {\rm{\Delta }}=1$ for $u=-\,1.5$ and $\hslash {\rm{\Delta }}=0.2$ for *u* = −2.1. For *u* = −2.1, the presence of a singularity so close to *ω* = 0 accounts for the non-monotonic behaviour shown in Fig. 4, displayed by *n*_*U*_ as *T* increases.

In Fig. 5 we plot the dependence of *K*_*β*_ (orange dotted line) and ${\tilde{\sigma }}_{xy}$ on frequency for two values of temperature *Tk*_*B*_ = 0.1 and *Tk*_*B*_ = 0.9 and for the two different values of the parameter *u* considered in Fig. 4. For *u* = −1.5 (green solid line) the model is in a topological phase at zero temperature (Ch = 1), while for *u* = −2.1 (black dashed line) the system is in a trivial zero-temperature phase (Ch = 0), but in close proximity to the critical value *u* = −2. As for the previous model, considered in Fig. 2, one can observe the appearance of van Hove singularities in transverse conductivity. Interestingly, for *u* = −2.1, one can observe the singularity at *ℏω* = 0.2, corresponding to the band gap of the model, which for *u* = −2.1 is given by *ℏ*Δ = 0.2. Clearly, as the model becomes critical, at *u* → −2, this peak will shift towards *ω* = Δ → 0. The presence of such a singularity for small values of *ω* explains the non-monotonic behaviour displayed by *n*_*U*_ in Fig. 4. For *T* ≪ 1, the distribution *K*_*β*_ is strongly peaked at *ω* = 0, and only the (trivial) static conductivity contributes to *n*_*U*_. As *T* increases, *K*_*β*_ broadens up, and picks up non-trivial contributions, mostly due to the singularity at *ω* = Δ.

This explanation of the non-monotonicity of *n*_*U*_’s behaviour is consistent with the interpretation in terms of thermal activation of the topological properties of the system. By considering formula for ${\tilde{\sigma }}_{xy}$ (see Eq. (33) in Methods), one realises that the peak of ${\tilde{\sigma }}_{xy}$ at the singular value ℏΔ = 0.2 carries information on the Berry curvature ${F}_{xy}^{B}$ in the Brillouin zone around at the band gap *ℏ*Δ. Close to criticality, this is the region that contributes the most to the overall value of zero-temperature Chern-number.

## Discussion

We have introduced the concept of *Uhlmann number* (see Methods), as a finite temperature generalisation of the Chern number. Beyond its mathematical and conceptual appeal, we have linked the *Uhlmann number* to directly measurable physical quantities, such as the dynamical susceptibility (see section Methods) and dynamical structure factor. We have shown that, in 2D translational invariant Fermionic systems, the above quantities can be straightforwardly measured through dynamical conductivity. This leads to a connection between Uhlmann number and transversal conductivity that may be thought as a finite-temperature generalisation of the (TKNN) formula. Moreover, these expressions highlights also a relation between the MUC, in the electric field parameters space, and *n*_*U*_. The latter shows that a non-trivial topology gives rise to an *incompatibility* condition in the parameter estimation problem of two orthogonal components of the electric field, due to the inherent quantum nature of the underlying physical system.

## Methods

### The Uhlmann number

The Uhlmann Geometric Phase is a generalisation of the Berry phase when the system is in a mixed state^35^. This generalisation relies on the idea of *amplitude* of a density operator $\rho \in  {\mathcal B} ( {\mathcal H} )$, which is defined as an operator *ω* satisfying $\rho =\omega {\omega }^{\dagger }$. Such a definition leaves a *U*(*n*) gauge freedom on the choice of *ω*, as any operator *ω*′ = *ωU*, with *U* unitary matrix, fullfils the same condition $\rho =\omega ^{\prime} {\omega ^{\prime} }^{\dagger }$. Let *ρ*_*λ*_ be a family of density matrices parametrized by $\lambda \in  {\mathcal M}$, with $\gamma \,:\,=\{\lambda (t)\in  {\mathcal M} ,t\in \mathrm{[0,}\,T]\}$ a smooth closed curve in a parameter manifold ${\mathcal M}$ and *ω*_*λ*_ the corresponding path of amplitudes. To reduce the gauge freedom, Uhlmann introduced a parallel transport condition on *ω*_*λ*_^35^. When this condition is fulfilled on a closed curve *γ*, the amplitudes at the endpoints of the curve must coincide up to a unitary transformation *ω*_*λ*(*T*)_ = *ω*_*λ*(0)_*V*_*γ*_, where *V*_*γ*_ is the holonomy associated to the path^35^.

The holonomy is expressed as ${V}_{\gamma }={\mathscr{P}}{e}^{i\oint A}$, where ${\mathscr{P}}$ is the path ordering operator, and $A={\sum }_{\mu }{A}_{\mu }d{\lambda }_{\mu }$ is the Uhlmann connection one-form, the non-Abelian generalization of the Berry connection. The Uhlmann connection is defined by the following ansatz^50,51^ ${\partial }_{\mu }\omega =\frac{1}{2}{L}_{\mu }\omega -i\omega {A}_{\mu }$, where *L*_*μ*_ are the Hermitian operators known as symmetric logarithmic derivative (SLD), and $({\partial }_{\mu }=\partial /\partial {\lambda }_{\mu })$ is the derivative with respect to a parameter in the manifold ${\mathcal M}$. The SLD is defined as the operator solution of the equation ${\partial }_{\mu }\rho =\frac{1}{2}\{{L}_{\mu },\rho \}$. The components of the Uhlmann curvature, the analogue of the Berry curvature, are defined as ${F}_{\mu \nu }={\partial }_{\mu }{A}_{\nu }-{\partial }_{\nu }{A}_{\mu }-i[{A}_{\mu },{A}_{\nu }]$. They can be understood in terms of the Uhlmann holonomy per unit area associated to an infinitesimal loop, ${F}_{\mu \nu }={lim}_{{\delta }_{\mu }{\delta }_{\nu }\to 0}i\frac{1-{V}_{\gamma }}{{\delta }_{\mu }{\delta }_{\nu }}$, where ${\delta }_{\mu }{\delta }_{\nu }$ is the area of the infinitesimal parallelogram spanned by the two independents direction ${\delta }_{\mu }{\hat{e}}_{\mu }$ and ${\delta }_{\nu }{\hat{e}}_{\nu }$.

The Uhlmann phase is defined as ${\phi }^{U}[\gamma ]={\rm{\arg }}\,{\rm{Tr}}[{\omega }_{\lambda \mathrm{(0)}}^{\dagger }{\omega }_{\lambda (T)}]$. The mean Uhlmann curvature^36^, defined as the Uhlmann phase per unit area for an infinitesimal loop, is given by

16 $${{\mathscr{U}}}_{\mu \nu }\,:\,=\mathop{\mathrm{lim}}\limits_{{\delta }_{\mu }{\delta }_{\nu }\to 0}\frac{{\phi }^{U}[\gamma ]}{{\delta }_{\mu }{\delta }_{\nu }}={\rm{Tr}}[{\omega }_{\lambda \mathrm{(0)}}^{\dagger }{\omega }_{\lambda \mathrm{(0)}}{F}_{\mu \nu }]\mathrm{.}$$

One can show that the MUC can be expressed in terms of the SLD in a very convenient way as

17 $${{\mathscr{U}}}_{\mu \nu }=\frac{i}{4}{\rm{Tr}}[\rho [{L}_{\mu },{L}_{\nu }]]\mathrm{.}$$

One can easy show that the MUC converges, in the pure state limit, to the Berry curvature ${F}_{\mu \nu }^{B}$.

The systems we study in this work are 2D translational invariant systems whose topology is characterised by the Chern number of the ground state, that is

18 $${\rm{Ch}}=\frac{1}{2\pi }{\int }_{BZ}{F}_{xy}^{B}d{k}_{x}d{k}_{y},$$

i.e. the integral over the first Brillouin zone (BZ) of the Berry curvature ${F}_{xy}^{B}={\partial }_{x}{A}_{y}^{B}-{\partial }_{y}{A}_{x}^{B}$, where ${A}_{\mu }^{B}=i\langle {\psi }_{k}|{\partial }_{\mu }|{\psi }_{k}\rangle$ is the Berry connection of the ground state. Here the parameter manifold is the BZ itself, i.e. ∂_*μ*_ = ∂/∂*k*_*μ*_, with *μ*, *ν* ∈ {*x*, *y*}.

Similarly, one can define the following quantity, the *Uhlmann number*, as the integral over the BZ of the MUC

19 $${n}_{U}=\frac{1}{2\pi }{\int }_{BZ}{{\mathscr{U}}}_{xy}d{k}_{x}d{k}_{y},$$

where, in analogy with eq. (18), ${{\mathscr{U}}}_{xy}$ is the MUC of Eq. (17), where the parameters {*λ*_*μ*_} are identified with the quasi-momenta *k*_*x*_ and *k*_*y*_. *n*_*U*_ is clearly a finite temperature generalisation of the Chern number, to which it converges in zero temperature limit. One easily sees that the MUC, and hence the *n*_*U*_, is gauge invariant, i.e. it does not depend on the gauge choice of the amplitude. Nonetheless *n*_*U*_ is not a topological invariant, and it is not always an integer as the Chern number is. In this work, we use *n*_*U*_ as an extension of the Chern number and we will link this quantity to physical proprieties of the systems.

In order to do this, let’s consider a 2D translational invariant systems, which may show non-trivial topology at zero temperature. The Hamiltonian of these systems can be cast in the following form,

20 $$ {\mathcal H} =\sum _{{\bf{k}}\in BZ}{\Psi }_{{\bf{k}}}^{\dagger }H({\bf{k}}){\Psi }_{{\bf{k}}},$$

where the first quantized Hamiltonian *H*(**k**), for two-band systems, is a 2 × 2 matrix. The latter can be written as $H({\bf{k}})={\varepsilon }_{{\bf{k}}}{\mathbb{1}}+{\overrightarrow{h}}_{{\bf{k}}}\cdot \overrightarrow{\sigma }$, where the ${\overrightarrow{h}}_{{\bf{k}}}$ is a 3D vector and $\overrightarrow{\sigma }$ are the Pauli matrices. ${{\rm{\Psi }}}_{{\bf{k}}}$ are Nambu spinors, which for two-band topological insulators are ${{\rm{\Psi }}}_{{\bf{k}}}\,:\,={({a}_{{\bf{k}}},{b}_{{\bf{k}}})}^{t}$, with ${a}_{{\bf{k}}}$ and ${b}_{{\bf{k}}}$ Fermionic annihilation operators of two different species of Fermions of the system. The Berry curvature assumes the following form,

21 $${F}_{xy}^{B}=\frac{1}{2}({\partial }_{x}{\hat{h}}_{{\bf{k}}}\times {\partial }_{y}{\hat{h}}_{{\bf{k}}})\cdot {\hat{h}}_{{\bf{k}}},$$

where $${\hat{h}}_{{\bf{k}}}={\overrightarrow{h}}_{{\bf{k}}}/|{\overrightarrow{h}}_{{\bf{k}}}|$$.

At thermal equilibrium, i.e. assuming a Gibbs state $\rho =\frac{{e}^{-\beta  {\mathcal H} }}{{\mathscr{Z}}}$, where $\beta =\mathrm{1/}{k}_{b}T$ is the inverse of the temperature and ${\mathscr{Z}}={\rm{Tr}}[{e}^{-\beta  {\mathcal H} }]$ is the partition function, the MUC ${{\mathscr{U}}}_{xy}$, calculated form Eq. (17) with respect to the parameters *k*_*x*_ and *k*_*y*_, reduces to the following simple expression

22 $${{\mathscr{U}}}_{xy}=\,\tanh (\frac{\beta |{\overrightarrow{h}}_{{\bf{k}}}|}{2}){\tanh }^{2}(\beta |{\overrightarrow{h}}_{{\bf{k}}}|)\cdot {F}_{xy}^{B}\mathrm{.}$$

In this form the MUC appears as a straightforward modification of the Berry curvature ${F}_{xy}^{B}$, to which it manifestly converges in the *β* → ∞ limit.

### Susceptibility and MUC

By using the linear response theory, we now derive a remarkable relation between the MUC, an inherent geometrical quantity, to a physically relevant quantity, the susceptibility. Let’s consider a system with a Hamiltonian ${ {\mathcal H} }_{0}$, perturbed as follows

23 $$ {\mathcal H} ={ {\mathcal H} }_{0}+\sum _{\mu }{\hat{O}}_{\mu }{\lambda }_{\mu },$$

where $\{{\hat{O}}_{\mu }\}$ is a set of observables of the system, and {*λ*_*μ*_} the corresponding set of sources. We are considering the system in thermal equilibrium, i.e. $\rho =\frac{{e}^{-\beta  {\mathcal H} }}{{\mathscr{Z}}}$, where ${\mathscr{Z}}={\rm{Tr}}[{e}^{-\beta  {\mathcal H} }]$ is the partition function. The dissipative part of the dynamical susceptibility, with respect to ${\hat{O}}_{\mu }$ is defined as:

24 $${\chi }_{\mu \nu }^{^{\prime\prime} }(t)=\frac{1}{2\hslash }{\langle [{\hat{O}}_{\mu }(t),{\hat{O}}_{\nu }]\rangle }_{0}$$

One can show that the Fourier transform of the dissipative part of the dynamical susceptibility has the following expression in the Lehmann representation

25 $${\chi }_{\mu \nu }^{^{\prime\prime} }(\omega ,\beta )=\frac{\pi }{\hslash }\sum _{ij}{({\hat{O}}_{\mu })}_{ij}{({\hat{O}}_{\nu })}_{ji}({p}_{i}-{p}_{j})\delta (\omega +\frac{{E}_{i}-{E}_{j}}{\hslash }),$$

where ${p}_{i}$’s are the eigenvalues of the density matrix in the Boltzmann-Gibbs ensemble, i.e. ${p}_{i}={e}^{-\beta {E}_{i}}/Z$, and ${E}_{i}$'s are the corresponding Hamiltonian eigenvalues. For thermal states, one can exploit the identity $\frac{{p}_{i}-{p}_{j}}{{p}_{i}+{p}_{j}}={\int }_{-\infty }^{+\infty }d\omega \,\tanh (\frac{\hslash \omega \beta }{2})\delta (\omega +\frac{{E}_{i}-{E}_{j}}{\hslash })$, which leads to the following relation between the ${\chi }_{\mu \nu }^{^{\prime\prime} }(\omega ,\beta )$ and the MUC,

26 $${{\mathscr{U}}}_{\mu \nu }=\frac{i}{\hslash \pi }{\int }_{-\infty }^{+\infty }\frac{d\omega }{{\omega }^{2}}{\tanh }^{2}(\frac{\hslash \omega \beta }{2}){\chi }_{\mu \nu }^{^{\prime\prime} }(\omega ,\beta ),$$

where the set of perturbations $\{{\lambda }_{\mu }\}$ in (23) plays the role of the parameters in the derivation of ${{\mathscr{U}}}_{\mu \nu }$. By means of the fluctuation-dissipation theorem^42^, one can further derive an expression for Eq. (26) in terms of the dynamical structure factor, ${S}_{\mu \nu }(\omega ,\beta )={\int }_{-\infty }^{+\infty }dt{e}^{i\omega t}{S}_{\mu \nu }(t)$ (i.e. the Fourier transform of the correlation matrix ${S}_{\mu \nu }(t)=\langle {\hat{O}}_{\mu }(t){\hat{O}}_{\nu }\mathrm{(0)}\rangle$), which reads

27 $${{\mathscr{U}}}_{\mu \nu }=\frac{i}{2\pi \hslash }{\int }_{-\infty }^{+\infty }\frac{d\omega }{{\omega }^{2}}{\tanh }^{2}(\frac{\hslash \omega \beta }{2})({S}_{\mu \nu }(\omega ,\beta )-{S}_{\nu \mu }(\,-\,\omega ,\beta ))\mathrm{.}$$

### Electrical conductivity and n_U_

Let’s assume now a 2D Fermionic system that presents translational invariance and let’s connect the above formulas to the Uhlmann number. In the quasi-momentum representation, the Hamiltonian reads ${ {\mathcal H} }_{0}={\sum }_{{\bf{k}}\in BZ} {\mathcal H} ({\bf{k}})$. If the system is perturbed by a time-dependent homogeneous electric field, the Hamiltonian is, up to first order,

28 $$ {\mathcal H} ={ {\mathcal H} }_{0}+{ {\mathcal H} }_{ext}={\int }_{BZ}{d}^{2}k( {\mathcal H} ({\bf{k}})-{{\bf{J}}}_{k}{\bf{A}}(t)),$$

where **J**_*k*_ is the electrical current density and **A**(*t*) is the potential vector. By exploiting standard linear response theory, one is able to link the conductivity, with the derivatives of the ${\mathcal H}$, as follows

29 $${\sigma }_{\mu \nu }^{^{\prime\prime} }(\omega ,\beta )=\frac{{e}^{2}}{{\hslash }^{2}}\sum _{i,j}{\int }_{BZ}{d}^{2}k\frac{\pi \delta (\omega +{\omega }_{ij})}{i\hslash \omega }({p}_{i}-{p}_{j})\times {({\partial }_{{k}_{\mu }} {\mathcal H} (k))}_{ij}{({\partial }_{{k}_{\nu }} {\mathcal H} (k))}_{ji}\,\,\mu ,\nu =x,y,$$

where ${\sigma }_{\mu \nu }^{^{\prime\prime} }$ is the dissipative part of the conductivity, defined as ${\sigma }_{\mu \nu }^{^{\prime\prime} }(\omega ,\beta )\,:=\frac{-i}{2}({\sigma }_{\mu \nu }(\omega ,\beta )+{\sigma }_{\nu \mu }(\,-\,\omega ,\beta ))$, in terms of the 2 × 2 conductivity tensor ${\sigma }_{\mu \nu }$. By using a procedure similar to that used to derive Eq. (26), we are able to calculate the following formula

30 $$\frac{1}{\pi }{\int }_{-\infty }^{+\infty }\frac{d\omega }{\omega }{\tanh }^{2}(\frac{\hslash \omega \beta }{2}){\sigma }_{xy}^{^{\prime\prime} }(\omega ,\beta )=-\frac{{e}^{2}}{2\pi \hslash }{n}_{U},$$

which links the dissipative part of the *dynamical transversal conductivity* ${\sigma }_{xy}^{^{\prime\prime} }(\omega ,\beta )$ to the Uhlmann number (Eq. (19)). Exploiting the symmetry properties of the conductivity with respect to *ω*, and plugging the Kramers-Kroing relations

31 $${\rm{Im}}[{\sigma }_{\mu \nu }(\omega ,\beta )]=-\frac{\omega }{\pi }{\mathscr{P}}{\int }_{-\infty }^{+\infty }\frac{{\rm{Re}}[{\sigma }_{\mu \nu }(\omega ^{\prime} )]}{{\omega }^{^{\prime} 2}-{\omega }^{2}}d\omega ^{\prime} ,\,\,\mu ,\nu =x,y,$$

into Eq. (30), yields eq. (7), which is displayed here for convenience,

32 $${n}_{U}\frac{{q}^{2}}{2\pi \hslash }=-{\int }_{-\infty }^{+\infty }d\omega {\tilde{\sigma }}_{xy}(\omega ,\beta ){K}_{\beta }(\omega \mathrm{).}$$

The above formula shows the dependence of ${n}_{U}$ only on ${\tilde{\sigma }}_{xy}(\omega ,\beta )$, the real, antisymmetric part of the dynamical transversal conductivity, which can be calculated, following a similar procedure as in^39^, as

33 $${\tilde{\sigma }}_{xy}(\omega ,\beta )\,:\,=\frac{{\sigma }_{xy}^{R}(\omega ,\beta )-{\sigma }_{yx}^{R}(\omega ,\beta )}{2}=-\frac{{e}^{2}}{\hslash }\frac{1}{{\mathrm{(2}\pi )}^{2}}{\mathscr{P}}{\int }_{BZ}{d}^{2}k\frac{{\omega }_{k}^{2}}{{\omega }_{k}^{2}-{\omega }^{2}}\,\tanh (\frac{\beta \hslash {\omega }_{k}}{2}){F}_{xy}^{B},$$

weighted by the kernel ${K}_{\beta }(\omega )$. The latter is defined as

34 $${K}_{\beta }(\omega )\,:\,=\frac{1}{{\pi }^{2}}{\int }_{-\infty }^{+\infty }d\omega ^{\prime} \frac{{\tanh }^{2}(\frac{\hslash \omega ^{\prime} \beta }{2})}{{\omega }^{^{\prime} 2}-{\omega }^{2}}=\{\begin{array}{ll}\frac{1}{i{\pi }^{3}}\frac{{{\rm{\Psi }}}^{\mathrm{(1)}}(\frac{1}{2}-\frac{i\hslash \beta \omega }{2\pi })-{{\rm{\Psi }}}^{\mathrm{(1)}}(\frac{1}{2}+\frac{i\hslash \beta \omega }{2\pi })}{\omega } & \omega \ne 0\\ -\frac{\hslash \beta }{{\pi }^{4}}{{\rm{\Psi }}}^{\mathrm{(2)}}(\frac{1}{2})=\frac{14\hslash \beta }{{\pi }^{4}}\zeta \mathrm{(3)} & \omega =0\end{array}$$

where ${{\rm{\Psi }}}^{(n)}(z)$ is the n-th poly-gamma function, defined as ${{\rm{\Psi }}}^{(n)}\,:\,=\frac{{d}^{n+1}}{d{z}^{n+1}}\,\mathrm{ln}\,{\rm{\Gamma }}[z]$, and $\zeta (z)$ is the Riemann zeta function. One can demonstrate that ${K}_{\beta }(\omega )$ is a probability density function over the frequency domain $\omega \in {\mathbb{R}}$, i.e. that ${K}_{\beta }(\omega )\ge 0$, $\forall \,\omega ,\beta \in {\mathbb{R}}$ and ${\int }_{-\infty }^{\infty }d\omega {K}_{\beta }(\omega )=1$. In particular,

$$\mathop{\mathrm{lim}}\limits_{\beta \to \infty }{K}_{\beta }(\omega )=\delta (\omega ),$$

showing that eq. (32) reduces to the TKNN formula in the zero-temperature limit.

Moreover, the probability distribution ${K}_{\beta }(\omega )$ is symmetric, peaked at $\omega =0$, and approximately non-vanishing only within a frequency band $\omega \in \{\,-\,{\rm{\Delta }}\omega ,{\rm{\Delta }}\omega \}$ of width ${\rm{\Delta }}\omega \simeq \frac{10}{\hslash \beta }$, which provides most of the contributions (about 92%) to the integral in eq. (32). This shows that *n*_*U*_ can be calculated as a weighted average of the real antisymmetric part of the dynamical transverse conductivity, with a dominant contribution due to the static conductivity, which grows as 1/*T* as temperature decreases.

## Conclusions and Outlook

In this work, we studied two prototypical models of TI and tested the behaviour of the Uhlmann number against the topological features of these models at non-zero temperature. We demonstrate the connection of the Ulhmann number to experimentally accessible quantities such as susceptibility and transverse conductivity, and derive a generalised TKKN formula. We investigated the implications of the above formula in both TI models. Our results suggests no indication of temperature-driven topological phase transitions, nor any actual phase transition at finite-temperature, in both models. Instead, we have found that the temperatures smooths out the transition between regions of zero-temperature topological order. Moreover, we observed an interesting non-monotonic behaviour of the *Uhlmann number n*_*U*_ in the QWZ model, which can be ascribed to a thermal activation of topological features for systems which are topologically trivial at zero temperature. We found that this effect is consistent with the appearance of the van Hove singularities in the dynamical conductivity. We foresee the possibility of extending the present analysis beyond uncorrelated models^52,53^.
